# Impact of experience of psychiatrists and psychiatry residents regarding electronic communication and social networking on internet use patterns: a questionnaire survey for developing e-professionalism in South Korea

**DOI:** 10.1186/s12909-019-1771-z

**Published:** 2019-11-08

**Authors:** Yeon Jung Lee, Jaeuk Hwang, Soyoung Irene Lee, Sung-Il Woo, Sang Woo Hahn, Steve Koh

**Affiliations:** 1 Department of Psychiatry, Soonchunhyang University College of Medicine, Seoul Hospital, Seoul, South Korea; 2 Department of Psychiatry, Soonchunhyang University College of Medicine, Bucheon Hospital, Bucheon, South Korea; 3 Department of Psychiatry, University of California, San Diego, California, USA

**Keywords:** Physician-patient relations, Communication, Electronic mail, Blogging, Psychiatry

## Abstract

**Background:**

The development of technology, novel communication, and social networking can positively or negatively affect the therapeutic alliance between patients and psychiatrists. We conducted this study to identify Internet use patterns of psychiatrists and psychiatry residents in South Korea and to provide basic data for developing e-professionalism.

**Methods:**

In this questionnaire survey included a total of 250 participants, of which 195 (78%) completed the questionnaire. Questions included demographics, use of email, web searches, personal and professional use of websites and social networking, and negative and positive experiences of electronic communication and social networking. We confirmed the correlation between experience and use patterns of psychiatrists’ electronic communication and social networking.

**Results:**

A total of 129 participants (66.2%) reported that they posted their personal or professional content online, 112 (57.9%) had received patients’ requests through electronic communication or social networking, and 120 (61.4%) had communicated with patients via electronic communication or social networking. In total, 170 participants (87.2%) reported that they were worried about the negative consequences of using electronic communication and social networking, and 180 (92.3%) indicated they were not educated about electronic communication or social networking.

**Conclusion:**

In order to reduce the negative effects of electronic communication and social networking, we need guidelines that are appropriate for the situation in South Korea. Furthermore, future research will need to identify and suggest solutions for negative experiences of electronic communication and social networking that may affect the relationship between patients and physicians.

## Background

Therapeutic alliances between patients and physicians are important factors affecting outcome and prognosis, particularly for psychiatrists [1]. The development of information and communication technology and digital media can have positive effects on the therapeutic alliance by providing medical information and web-based treatment, which contribute to patients’ convenience [2, 3]. While the in-person relationships will undoubtedly remain the core of medical and psychiatric practice, developing online relationships via email, text messaging, and telephone have been observed to greatly improve access and interactions during different times and in different places [4]. However, social networking, including social media and electronic communication, can make the distinction between publicity and privacy unclear, impairing the professionalism of psychiatrists, interfering with the formation of therapeutic relationships between patients and psychiatrists, and threatening the privacy and safety of psychiatrists [5–7].

Many medical students and physicians are using the Social Networking Services (SNS). According to Thompson [8], many medical students and residents (44.5%) had a Facebook account, and 62.7% kept their Facebook account public. In Gupta et al.’s study of medical students in India, 477 (78.1%) from a sample of 611 students had public Facebook profiles. These public profiles contained identifiable profile pictures (80.3%), fields of study (51.6%), institutions (86.2%), and friend lists (88.7%) [9]. Moubarak et al. [10] reported that many residents and fellows (73%) had a Facebook account, out of which 52% connected to Facebook more than once per day [9]. In other studies [6, 11], residents and physicians reported that they received friend requests from patients or patients’ families. Many physicians and medical students are already communicating with patients and people related to these patients through SNS, video, e-mail, and EMRs; these web-based and mobile tools are constantly evolving [4, 12]. Therefore, to avoid being overwhelmed by changes in the environment, psychiatrists and related mental health practitioners should make efforts to develop hybrid doctor-patient relationships, both in-person and online.

Cain et al. [13] defined e-professionalism as reflecting the paradigm of traditional experts implemented through digital media, because social networking usage influences medical professionalism, ethics, and privacy protection. Cooke et al. [14] reported that e-professionalism should be included in medical education because the formation of professional identity and the development of professional values and beliefs that influence behavior should be the main focus of medical education. E-professionalism in the United States, Australia, and New Zealand is a subject of interest and is being taught as a part of medical education [6, 13, 15, 16]. Although physicians, including psychiatrists, in South Korea are interested in and have considered these issues, there are no existing guidelines or educational information on this topic [17, 18]. Therefore, it is necessary for South Korea to establish e-professionalism in line with the current situation of the information age.

Our aim for this study was to identify the patterns of use of electronic communication and social networking among psychiatrists and psychiatry residents of South Korea and to examine the impact of their experience on usage patterns, with a goal of providing basic data for the formation of Korean e-professionalism. First, we assessed the electronic communication and social networking usage patterns of psychiatrists and psychiatry residents. Second, we assumed that psychiatrists and psychiatry residents’ experiences of the Internet would affect usage behavior. To confirm these, we assessed (1) whether restrictive access to personal information on the Internet would be affected by positive or negative experiences of the use of electronic communication and social networking; and (2) whether psychiatrists and psychiatry residents with negative social networking experiences would be more concerned about the negative impact of electronic communication, social networking, and self-googling.

## Method

### Participants

This study was conducted with psychiatrists and psychiatry residents of South Korea; the study method of Koh et al. [6] was followed, after obtaining their approval. The study was conducted from June 1 to December 31, 2016, with psychiatrists and psychiatry residents who attended various academic conferences. In total, 250 questionnaire surveys were distributed to participants who attended the autumn and winter conference of the Korean Neuropsychiatric Association and its sub-associations, and 201 (80.4%) were collected. Data from 195 questionnaire surveys were analyzed, excluding six questionnaires with no responses. The Institutional Review Board/Ethics Committee of Soonchunhyang University of South Korea approved the study protocol, which was in accordance with the Declaration of Helsinki (2016–04–020-002). We explained the purpose and method of the study to the participants in entirety and obtained their written consent.

### Instrument

In this study, a self-report questionnaire survey developed by Koh et al. [6] was used. Koh et al.’s survey was developed to collect data about the patterns of use of electronic communications and social media among practicing psychiatrists, and to establish a conceptual framework for developing professional guidelines. Although this questionnaire survey is not standardized, Koh et al. reported useful results, and the use of this questionnaire survey allowed for the comparison of data between American and Korean psychiatrists. The questionnaire survey content used in this study was the same except that the demographic data was transferred from the back to the front of the questionnaire survey. The questionnaire survey included questions on demographics, use of email, web searches, and personal and professional use of websites and social networking. Structured and open response questions addressed ethical, legal, and safety concerns as well as the negative or positive consequences of electronic communication and social networking. The 5-point Likert scale terms used in the questionnaire survey were defined as follows: never (0%), rarely (< 10%), sometimes (10–50%), routinely (50–80%), and almost always (> 80%).

Answers to the three questions, “Do you restrict access to personal information on the Internet (privacy settings)?,” “Have you experienced positive outcomes from the use of electronic communications and social networking?” and “Have you experienced negative outcomes from the use of electronic communications and social networking?,” were reclassified as follows to evaluate our hypothesis: never and rarely as “No” (not limited or inexperienced), and sometimes, routinely, and almost always as “Yes” (limited or experienced).

### Statistics

First, a descriptive statistical analysis was performed to calculate the frequencies and percentages of general characteristics and social media usage patterns of the participants. For continuous variables, mean and standard deviation (SD) were used as summary statistics. General characteristics were gender, age, and acquisition of the participants. The questionnaire survey about usage patterns included questions that enquired whether participants googled themselves or patients, posted personal or professional content online, and their positive or negative experiences of electronic communication and social networking. Second, logistic regression was performed to determine 1) whether restrictive access to personal information on the Internet would be affected by positive or negative experiences of the use of electronic communication and social networking; 2) whether subjects with negative experiences in electronic communication and social networking would be more concerned than those with positive experiences about the negative impact of electronic communication and social networking; and 3) whether having negative experiences of electronic communication and social networking would affect self-googling more than positive experiences. All statistical analyses were performed using STATA 15, with a statistical significance of *p* < 0.05.

## Results

### Demographic characteristics

Of the 195 participants, 110 (56.4%) were men and 85 (44.1%) were women. Participants represented a wide range of ages (25–64 years, mean 35.4 years, SD 7.4 years). In total, 116 (59.5%) were psychiatrists, and 79 (40.5%) were psychiatry residents being trained to become psychiatrists.

### Use of electronic communication and social networking among psychiatrists

Of the 195 participants, 69.2% rarely or never used online search engines to determine what information was publicly available about them. In total, 31.7% of participants had, at some time, searched for online information about their patients. The vast majority rarely or never communicated with patients via text messaging (91.3%) or e-mail (94.8%). Of those participants who communicated with patients via text messaging, 71.8% had never obtained permission from the patients. Of those participants who communicated with patients via email, 63.1% had never obtained permission from the patients (Table 1).

**Table 1:** Use of electronic communication by psychiatrists and psychiatry residents (*N* = 195)

Questions-How many have:	Almost always	Routinely	Sometimes	Rarely	Never
Googled themselves	3.6%	0	27.2%	44.1%	25.1%
Googled patients	0	1.5%	5.6%	24.6%	68.2%
Posted online content about themselves	1.0%	4.6%	19.5%	37.4%	37.4%
Restricted online personal information	40.5%	30.3%	25.1%	3.6%	0.5%
Texted patients	0	0.5%	8.2%	24.6%	66.7%
E-mailed patients	0	1.0%	4.1%	25.6%	69.2%

**Fig. 1:**
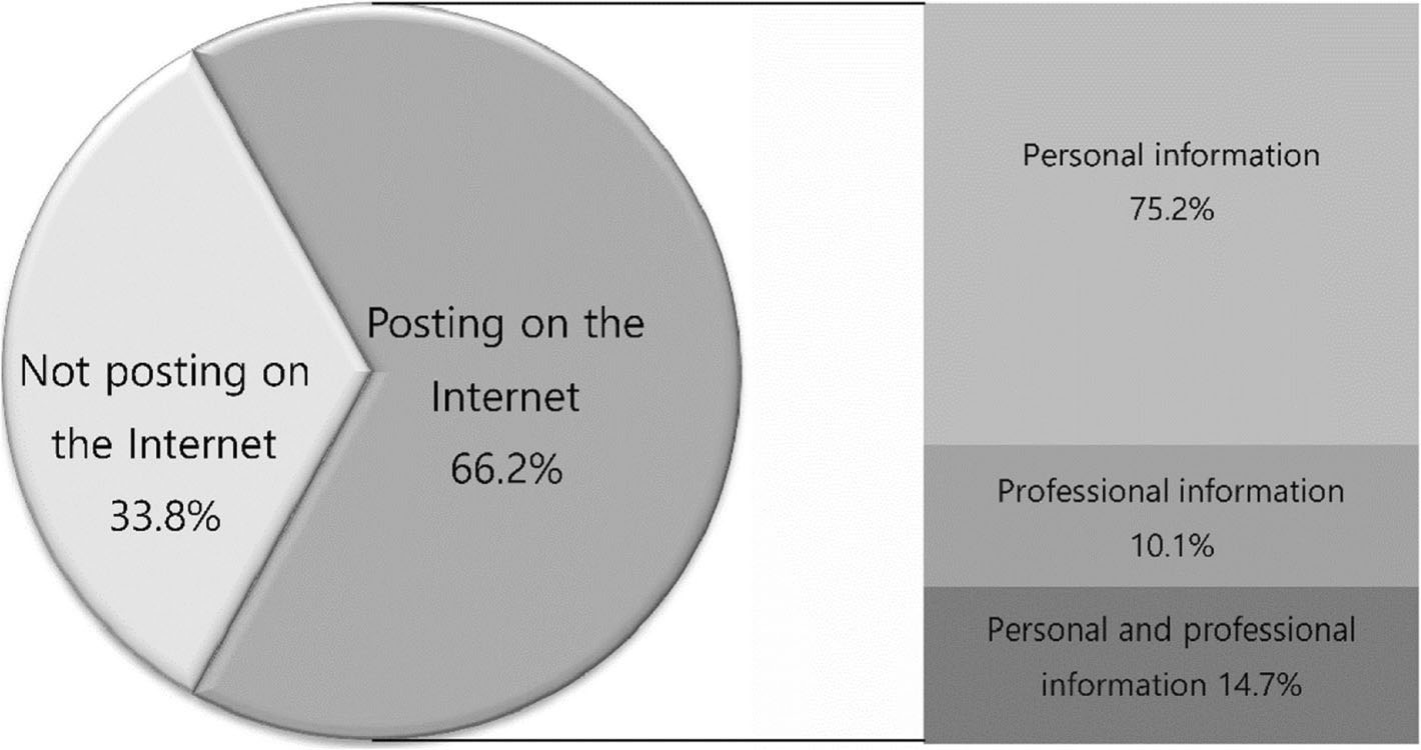
The use of social networking by Korean psychiatrists and psychiatry residents

In total, 66.2% posted online content, 75.2% posted personal information, and 14.7% posted personal and professional information (Fig. 1).

### Factors related to social communication of psychiatrists and psychiatry residents

Many participants (64.1%) sometimes, routinely, or almost always experienced positive outcomes from the use of electronic communication and social networking. They reported that maintaining relationships and exchanging information and knowledge were positive outcomes. On the other hand, 23.1% participants sometimes, routinely, or almost always experienced negative outcomes (Table 2). They reported that lack of privacy, contact from unwanted people, transmission of unfounded information, hacking, unnecessary haggling, and so on were negative outcomes. Many participants (57.9%) had received patient requests via e-mail or social networking (Table 2). Only 7.7% of participants indicated that they were educated about social networking/electronic communication.

**Table 2:** Experiences of using electronic communication and social networking (*N* = 195)

Question		Yes	No
Positive experiences from the use of electronic communication and social networking		125 (64.1%)	70 (35.9%)
Negative experiences from the use of electronic communication and social networking		45 (23.1%)	150 (76.9%)
Concerns about negative effects of electronic communication and social networking		170 (87.2%)	25 (12.8%)
Educational experiences about social networking/electronic communication		15 (7.7%)	180 (92.3%)
Experience of patient requests	No	82 (42.1%)	
	Yes	113 (57.9%)	
	Type of request	Email contact	78 (40.0%)
		Personal posting	40 (20.51%)
		Professional posting	82 (42.05%)

The restriction of personal information on the Internet was statistically significantly affected by positive and negative experiences of electronic communication and social networking. In addition, those with negative experiences were more concerned about negative effects of electronic communication, social networking, and self-googling (Table 3).

**Table 3:** Internet usage patterns of Korean psychiatrists and psychiatry residents regarding electronic communication and social networking

		Restricted access			Concerns about negative effects			Self-googling		
		OR.	95% CI	*P* value	OR.	95% CI	*P* value	OR.	95% CI	*P* value
Experiences of electronic communication and social networking	Positive experience	2.370	.129–1.596	.021^**^	1.095	−.781–.964	.837	1.545	−.253–1.122	.215
	Negative experience	2.344	.147–1.556	.018^**^	3.951	−.967–2.867	.071^*^	2.832	.345–1.736	.003^***^

*OR* Odds ratio, *95% CI* Confidence Interval, * *p* < 0.5, ** *p* < 0.05, *** *p* < 0.01

## Discussion

In this study, we assessed Internet use patterns of psychiatrists and psychiatry residents in South Korea based on their electronic communication and social networking, and identified whether positive or negative experiences of electronic communication or social networking affected these patterns. The main findings were as follows: (1) 66.2% of psychiatrists and psychiatry residents posted online content, and 75.2% posted personal information; (2) 23.1% experienced negative outcomes; (3) only 7.7% indicated that they were educated about electronic communication/social networking; (4) only 40.5% of psychiatrists and psychiatry residents almost always restricted online personal information; (5) many (57.9%) had received patient requests via e-mail or social networking; and (6) those with negative experiences were more concerned about negative effects of electronic communication, social networking, and self-googling.

This study found that many psychiatrists and psychiatry residents in South Korea were posting on the Internet and receiving patient requests through e-mail communication and social networking. We found that 75.2% of them posted personal information, but only 40.5% of psychiatrists and psychiatry residents almost always restricted online personal information. In a previous study on medical students [8], 37.5% did not use privacy settings on social networking. In another study [19], medical students reported that they had posted unprofessional content (e.g., sexually suggestive pictures or comments, profanities, discriminatory language, pictures of themselves or peers engaging in drug use). While previous studies had focused on medical students only, this study focused on clinical physicians as well; moreover, the sample of this study was more familiar with internet usage than those in previous studies. However, as had been observed in previous studies, participants in this study also posted personal information publicly.

This study found that many (57.9%) participants had received patient requests via e-mail or social networking; this percentage was significantly higher than that of previous studies. In a stratified mail survey by Bosslet et al. [11], 2% of medical students, 7.8% of resident physicians, and 34.5% of practicing physicians had received a friend request from a patient or a patient’s family member. In total, 19.4% of medical students believed that it is ethically acceptable for physicians to interact with patients through personal online social networking sites [11]. The difference between the previous studies and this study is that we aimed at clinicians and that our sample included a larger number of internet users. The exact number of medical students who communicate with patients or patients’ family members has not been confirmed, but it is estimated to be sizable. These behaviors can reduce their medical professionalism or threaten their career in the future. Moreover, unprofessional online behavior by medical students or physicians may undermine the public’s trust in the medical profession as a whole [15]. Therefore, medical students or physicians should be educated about this before they commence clinical practice.

In this study, 87.2% of psychiatrists and psychiatry residents reported that they were worried about negative outcomes of electronic communication/social networking, and 23.1% of those involved in this study had experienced such negative outcomes. A significant number of participants reported negative views. However, telepsychiatry, which is psychiatry-focused telemedicine originated with institutionally based videoconferencing, has not yet been introduced in South Korea, even though it is already over 50 years old [4]. While mental health care providers have shown lower satisfaction and concern for telepsychiatry, as compared to allied health providers and patients, mental health care professionals and patients consider it an acceptable delivery method [4, 20]. Telepsychiatry reported similar clinical outcomes and satisfaction with in-person care in depression, anxiety, PTSD, panic disorder, and attention deficit/hyperactivity disorder [20]. Text-messaging interventions aimed at medication adherence have been effective among patients taking psychotic medications to manage schizophrenia [21]. In this study, only 7.7% indicated that they were educated about electronic communication/social networking. The lack of concepts and information on telepsychiatry may still be a major concern.

Koh et al. [6] proposed that comprehensive guidelines on electronic communication and social networking for psychiatrists should address four key areas or “lenses”: 1) treatment frame, 2) patient privacy/confidentiality, 3) medico-legal concerns, and 4) professionalism. At the beginning of treatment, they emphasize that it is necessary to google patients and to provide information to patients and their families through e-mail after obtaining consent in writing for legal reasons. In particular, they insist on considering the impact of “dual relationships” when befriending a patient through online social media, and establishing a clear distinction between professional and personal online sites.

The Council on Ethical and Judicial Affairs of the American Medical Association (AMA) [15] and the Royal Australian and New Zealand College of Psychiatrists (RANZCP) Congress [16] emphasize privacy issues similar to those reported by Koh et al. [6] in the use of social media. The AMA also states that physicians are obligated to advise peers to remove content that violates professionalism and to take appropriate action. Content on the Internet must be posted with caution as it has a wide impact, and the behavior of one individual can negatively affect all physicians [15]. RANZCP Congress recommends that, in case of a patient emergency and lack of access to the Internet, a protocol using a contract at the time of treatment must be specified.

In this study, 57.9% of psychiatrists had received patient requests via e-mail or social networking regarding medical questions, personal contact, or friend requests. Although more psychiatrists had received these requests, 16.9% of residents reported receiving a friend request or e-mail from patients. According to the American Medical Informatics Association (AMIA), patients need to know the turnaround time, and how frequently the physician goes online to respond to patients [22]. The guidelines recommended that physicians should refrain from expressing uncomfortable feelings, and that they must maintain a professional tone. If psychiatrists receive friend requests from patients or their family members via social networking, they should consider whether to accept by separating their personal and public sites. In addition, when psychiatrists accept friends through a personal site, they should consider the privacy of other psychiatrists or colleagues. This is because personal information is disclosed without the consent of other physicians and colleagues who are friends with the psychiatrist. Thus, it is necessary for South Korean psychiatrists to create guidelines for electronic communication and social networking and to provide education on these guidelines.

In this study, psychiatrists and psychiatry residents with negative experiences of electronic communication and social networking were more concerned about negative effects and self-googling. While the former result may be expected, the latter is a subject of interest. Previous studies have shown that positive verbal feedback stimulates and enhances intrinsic motivation and develops a more competent feeling than negative verbal feedback [23, 24]. Our results are different from that of previous studies as it is assumed that the negative experience on the Internet may have a slightly different mechanism. Therefore, further systematic studies are needed to confirm the exact effect.

This study had a number of limitations. First, psychiatrists and psychiatry residents participating in this study did not represent all psychiatrists in South Korea because they were limited to psychiatrists who participated in psychiatric conferences. Second, the mean age was relatively young at 35.4 years. As young psychiatrists are more familiar with social networking media, results from our sample may have overestimated the actual usage of social media among psychiatrists. Third, in this study, only positive or negative experiences on the SNS were identified and journals were not confirmed. Among the journals listed in South Korea’s social science citation index, the journal scored highest journal was the Psychiatry Investigation [25]. In addition, this study did not elaborate on negative experiences in electronic communication and social networking, which could have an impact on future doctor-patient relationships. Altmetrics may also have an impact on doctor patient relationship; therefore, subsequent studies should also include an analysis of Altmetrics in the psychiatry area. Future studies will need to analyze the social networking usage of psychiatrists by increasing the sample size of various age groups.

## Conclusions

This study confirmed usage patterns of e-mail communication and social networking of psychiatrists and psychiatry residents in South Korea. Standards for privacy protection and online doctor-patient relationship have not yet been established. In order to develop the e-professionalism of psychiatrists in South Korea, guidelines for electronic communication and social networking are needed. In addition, it is necessary to confirm the influence of negative experiences in electronic communication and social networking on doctor-patient relationship through more systematic research in the future.

## Data Availability

The datasets analyzed during this study can be obtained through the corresponding author on reasonable request.

## Abbreviations

AMA: The American Medical Association
AMIA: American Medical Informatics Association
SNS: Social networking services
